# Hypoglycemic Ability of Sericin-Derived Oligopeptides (SDOs) from *Bombyx mori* Yellow Silk Cocoons and Their Physiological Effects on Streptozotocin (STZ)-Induced Diabetic Rats

**DOI:** 10.3390/foods13142184

**Published:** 2024-07-11

**Authors:** Chainarong Tocharus, Manote Sutheerawattananonda

**Affiliations:** 1 Department of Anatomy, Faculty of Medicine, Chiang Mai University, Chiang Mai 50200, Thailand; chainarongt@hotmail.com; 2 School of Food Technology, Institute of Agricultural Technology, Suranaree University of Technology, Nakhon Ratchasima 30000, Thailand

**Keywords:** hypoglycemic, diabetes, sericin, bioactive peptide, physiological

## Abstract

Patients with diabetes require daily medication to maintain blood sugar levels. Nevertheless, the long-term use of antidiabetics can lose efficacy and cause degeneration in some patients. For long-term diabetes care, integrating natural dietary foods and medicine is being considered. This study investigated the impact of SDOs on blood sugar levels and their physiological effects on diabetic rats. We induced diabetes in male Wistar rats with STZ (50 mg/kg) and then administered an oral glucose tolerance test to determine the SDO dosage comparable to glibenclamide. The rats were divided into nine groups: normal, diabetic, and diabetic with insulin (10 U/kg), glibenclamide (0.6 mg/kg), bovine serum albumin (BSA; 200 mg/kg), soy protein isolate (200 mg/kg), or SDOs (50, 100, and 200 mg/kg). Diabetic rats administered SDOs had a higher body weight and serum insulin but a lower blood sugar than diabetic control rats. Biochemical assays indicated lower AST/SGOT, ALT/SGPT, BUN, and triglycerides but higher HDL in the SDO groups. Immunohistochemistry showed that SDOs reduced damaged islet cells, increased beta-cell size, and improved insulin levels while decreasing alpha cell size and glucagon. The vascular effects of SDOs were like those of normal control treatment and insulin treatment in diabetic rats. SDOs, a yellow silk protein, show potential for long-term diabetes care.

## 1. Introduction

In 2045, 783.2 million people will have diabetes mellitus (DM) [1]. Diabetes in Thailand is projected to reach 5.454 million people by 2030 [2]. Impairment in insulin secretion from pancreatic beta cells, insulin deficiency due to beta-cell destruction, or insulin resistance within the target tissue can cause diabetes [3]. Diabetes is a multifactor metabolic syndrome that tends to have complications arising from alterations in the pathology of macrovascular and microvascular blood vessels, encompassing conditions such as coronary vascular disease, cerebrovascular disease, peripheral vascular disease, diabetic retinopathy, diabetic nephropathy, diabetic neuropathy, and diabetic ulcers [4]. Type 2 diabetes mellitus (T2DM) patients tend to develop atherosclerosis from the accumulation of adipose and fibrous tissue, leading to the thickening of arterial walls and causing coronary vascular diseases (CVDs) [5]. CVDs affect 32.2% of T2DM patients and account for 50% of mortality [6]. Diabetes and CVDs are correlated with combined parameters such as overweight, high cholesterol, a particular diet, physical activity, walking ability, and air pollution (nitrogen dioxide (NO_2_) and PM 2.5) more than genetics [1,7,8,9]. Overweight and high cholesterol are strong risk factors for developing T2DM, causing inflammation that leads to prediabetes and diabetes [6,8].

T2DM patients are treated with oral hypoglycemic medications to restore normal blood sugar and lipid levels. These medicines can be classified into four groups: α-glucosidase inhibitors (acarbose and voglibose) [10], sulfonylureas (tolbutamide, glibenclamide, and glimepiride) [11], biguanides (metformin) [11], and thiazolidinediones (rosiglitazone and pioglitazone) [12]. The Diabetes Prevention Program (DPP) of the National Institute of Health recommends metformin for high-risk groups with prediabetes [11]. A new class of diabetes drugs has been developed: SGLT-2 inhibitors, which allow the kidneys to excrete more glucose; GLP-1 agonists, which increase insulin secretion; and iminosugars, such as 1-deoxynojirimycin, found in mulberry, which can inhibit gut glucosidase [13]. However, these drugs have limitations such as metformin’s poor permeability to cell membranes [14], resulting in slow and incomplete absorption [15]; thiazolidinediones’ short half-life (3–5 h), leading to low bioavailability and decreased therapeutic efficacy [14]; and various mild to serious side effects, negatively impacting patient adherence and compliance [16]. Prospective strategies for long-term T2DM management may include multi-target compounds [17]. This is where natural compounds like whey, soy, and other natural compounds with different action pathways can be beneficial and represent a topic of interest for long-term studies to evaluate their efficacy on various T2DM and glycemia parameters. These proteins and natural compounds reduce hyperglycemia to levels comparable to those achieved with drugs such as sulfonylureas.

Dietary proteins like whey and soy have been reported to have hypoglycemic properties. Several cohort studies have reported a significant inverse relationship between dairy product intake and the risk of T2DM [18,19], whereas the consumption of eggs and fish does not exhibit such a relationship [20,21]. Whey has been shown to decrease postprandial glycemia and induce insulin secretion in obese, pre-diabetic, and T2DM patients [1]. This could be used as a complementary therapeutic method for managing blood sugar levels [1]. Whey protein as low as 15 g when consumed shortly before meals can stimulate insulin release, improve postprandial glycemia (−13%), and increase satiety in T2DM groups (*p* < 0.05) [22]. Moreover, whey protein may have beneficial effects on some metabolic disorders and lower cardiovascular risk factors [23]. Metabolic disorders resulting from hyperglycemia, hypertension, excess body fat around the waist, and abnormal cholesterol or triglyceride levels can increase the risk of diabetes, heart disease, and stroke [24]. Whey’s insulinotropic effect is associated with branched-chain amino acids (BCAAs), especially leucine, isoleucine, valine, lysine, and threonine. These amino acids induce insulin secretion, with leucine reportedly having the greatest acute effect [25,26,27]. Leucine raises glutamate dehydrogenase activity in beta cells, increasing Krebs cycle activity, which subsequently leads to increased insulin production [28].

Soy protein has also been found to lower postprandial hyperglycemia [29]. Unlike animal proteins, soy has no cholesterol and is low in methionine and branched-chain amino acids (BCAAs) [30]. BCAAs, especially leucine and its derivatives, are important for muscle function [31]. High levels of BCAAs in the blood can cause abnormal metabolism, leading to obesity, insulin resistance, T2DM, cancer, and heart failure [32]. Studies have found that a low-BCAA diet can increase the survival rate of mice with premature aging, delay frailty, and promote metabolic health [33,34]. The same studies suggested that restricting dietary BCAAs can increase the health and life span of mice. Polyunsaturated fatty acids, lecithin, and stigmasterol, in which soy is rich, have shown the ability to reduce total cholesterol and low-density lipoprotein cholesterol levels, to improve diabetes, and to protect cardiovascular health [35]. A meta-analysis demonstrated a significant negative association between soy consumption and the incidence rate of T2DM. Soy protein and flavonoids are important parts of the soy active ingredients and could reduce the risk of CVDs and diabetes through their antioxidative and anti-inflammatory properties [36]. Several clinical and basic studies also support this finding [37,38,39]. Stigmasterol, a common phytosterol in soybeans, improves blood glucose and induces beta-cell regeneration by improving GLUT4 translocation and insulin resistance [40]. Soy consumption will play an important role in T2DM and CVD prevention strategies.

Yellow silk sericulture (*Bombyx mori*), a traditional practice in the northeastern region of Thailand, produces silk sericin protein as a byproduct. The yellow silk cocoons consist of 90% live cocoon and 10% silk thread that has two types of proteins: 65% fibroin and 35% sericin. During the silk-reeling process, sericin is extracted and discarded in wastewater. Sericin, a protein produced by the silk glands of silkworms alongside liquid fibroin, contributes to the formation of silk cocoons [41]. Due to its adhesive properties, sericin functions as a bonding agent that reinforces the structure of the cocoon by adhering two fibroin strands together [42]. Interestingly, sericin has 18 essential amino acids, mostly serine, glycine, and glutamine, but is low in BCAAs, like soy, and has antioxidant and anti-tyrosinase properties that can be used in biomaterials and cosmetics [43,44]. Sericin can lower cholesterol levels in rats fed high-fat diets [45] and reduce the risk of colon cancer [46], possibly due to its antioxidant activity and cholesterol-binding ability, which are like those of dietary fibers. However, the mechanisms through which sericin exerts its anti-cancer effects are unknown. When low and high molecular weights of sericin were compared, the lower one exhibited higher anti-proliferative effects than the higher one in both human colorectal cancer SW480 cell and normal colonic mucosal FHC cell viability tests. SDOs comprise small peptides that are highly water-soluble and readily absorbed by the body, which could increase their efficacy. The physicochemical and biochemical properties of protein-derived oligopeptides are different from those of polypeptides, which tend to change their internal structure to more stable forms [47]. Oligopeptides may possess the ability to have specific receptor-like structures, thereby functioning as regulators of other proteins [48]. They can be derived from various sources, such as plant proteins, e.g., soybeans and corn, and animal proteins, e.g., bovine serum and shrimp shells [49]. Notably, they are devoid of bacterial and fungal contaminants [50]. Research has indicated that oligopeptides obtained from marine salmon skin (OMSS) [50] and *Momordica charantia* L. Var. (Ser. protein hydrolysate) can lower blood sugar levels [51]. Silk protein hydrolysate derived from white silk cocoons has also demonstrated blood-sugar-reducing effects [52,53]. A previous investigation has shown that sericin-derived oligopeptides (SDOs) from yellow silk have dual-functional DPP-IV and ACE-inhibitory effects in vitro [54]. Nevertheless, there are currently few animal studies exploring the impact of oligopeptides derived from the sericin protein of the yellow silk cocoon, *Bombyx mori*, on blood sugar levels. The objective of this study was to investigate the optimal dosage of SDOs for reducing blood glucose in diabetic rats and assess their effects on blood glucose control and biological functions.

## 2. Materials and Methods

### 2.1. Preparation of Sericin-Derived Oligopeptides (SDOs)

SDOs were prepared following the protocol of Sutheerawattananonda et al. [55]. Yellow silk cocoons of the *Bombyx mori* Nang Noi Srisaket-1 variety purchased from Queen Sirikit Sericulture Centers, Chaiyaphum, Thailand, were autoclaved for 30 min to dissolve sericin protein. The sericin-rich protein solution was filtered through a cheesecloth to separate the extracted cocoons from the liquid part. The sericin solution obtained was subjected to enzymatic hydrolysis by protease (from *Bacillus* sp., 16 U/g, EC no. 2327522, Sigma, St. Louis, MO, USA). A 1 mL aliquot of protease enzyme solution (0.01 unit/mL protease enzyme in 0.036 M calcium chloride (CaCl_2_) solution at a 1:1 volumetric ratio) was added to 300 mL of the obtained sericin solution and incubated under shaking conditions at 37 °C for 1 h. The solution was then heated to 90 °C for 15 min to stop the enzymatic activity and cooled to room temperature before centrifugation at 9500× *g* for 15 min at 4 °C to separate the solid portions. Oligopeptides with a molecular weight lower than 5 kDa were separated from larger oligopeptides by ultra membrane filtration using a hollow-fiber membrane with a 5000 molecular weight cut-off (MWCO) (GE Healthcare Bio-Sciences AB, Uppsala, Sweden). The oligopeptide solution obtained was freeze dried and kept in a sealed container at room temperature until use. For oral feeding to the animals, the SDO powder was dissolved in distilled water to form a solution according to the experimental concentrations.

### 2.2. Animals and Treatments

The National Laboratory Animal Center at Mahidol University, Salaya, Nakhon Pathom, Thailand, provided 54 male Wistar rats weighing 180–200 g at 8 weeks old. All animals were kept under a 12:12 h light–dark cycle at a temperature of 25 ± 1 °C. The animals were given free access to rodent diets (C.P. Company, Bangkok, Thailand) and reverse osmosis (RO) water. The experimental protocol was approved by the Research Ethics Committee of the Faculty of Medicine, Chiang Mai University, Thailand, under approval number 15/2555, and followed Chiang Mai University’s guide for the care and use of laboratory animals. On the completion of feeding, rats were sacrificed by administering an overdose of 50 mg/kg body weight (BW) (intraperitoneal, i.p.) of thiopental sodium (THIOPENTAL, Biopharma, Thailand). Blood was immediately collected by cardiac puncture; subsequently the spleen, thymus, liver, lung, and kidney were aseptically removed and weighed.

#### 2.2.1. Diabetic Induction in Animals

Diabetes was induced in six rats by administering STZ at a single dose of 50 mg/kg in 0.2 mL citrate buffer intravenously at the base of the tail. The control (*n* = 6) received only 0.2 mL sterile citrate buffer; after one week, blood glucose was measured by drawing blood from rats. Glucose levels in the blood were then determined using a glucose test kit (ACCUCHEK, Roche Laboratories, Pharma, Mannheim, Germany). Normal rats and rats with hyperglycemia of 300 mg/dL or higher were used for subsequent experiments.

#### 2.2.2. Study of Acute Effects and Optimum Dose of SDOs on Blood Glucose Lowering in Diabetic Rats with Acute Hyperglycemia by Oral Glucose Tolerance Tests (OGTTs)

After an overnight fast, rats were divided into six groups (*n* = 6) as follows: group 1 normal rats and group 2 diabetic rats received distilled water, and group 3 diabetic rats received glibenclamide (Sigma, St. Louis, MO, USA) at a concentration of 0.6 mg/kg [56]. Group 4, 5, and 6 received SDOs at concentrations of 50, 100, and 200 mg/kg, respectively. In this experiment, each group of rats received the aforementioned substances by oral gavage. After 30 min, a glucose solution was fed at a concentration of 5 g/kg [57]. Blood was collected from the tail vein to record blood glucose levels before administering the tested substances and the glucose solution (−30 and 0 min, respectively). The blood glucose was then measured 30, 60, 90, and 180 min after glucose administration [58].

### 2.3. Study of Biological Effects of SDOs on Diabetic Rats

The rats were divided into nine groups (*n* = 6). The normal control group (Gr. 1) received 0.2 mL citrate buffer, and blood glucose levels were in between 80 and 120 mg/dL. For the diabetic rats with a blood glucose level > 300 mg/dL, they were separated into a diabetic control group that received distilled water (Gr. 2), a diabetic group that received 10 U/kg insulin (Eli Lilly Asia Inc., Thailand branch, Bangkok, Thailand) (Gr. 3), a diabetic group that received 0.6 mg/kg glibenclamide (Gr. 4), a diabetic group that received 200 mg/kg bovine serum albumin (BSA) (Sigma, St. Louis, MO, USA) (Gr. 5), a diabetic group that received 200 mg/kg soy protein isolate (SPI) (Krungthepchemi, Bangkok, Thailand) (Gr. 6), and diabetic groups that received 50, 100, and 200 mg/kg SDOs (Gr. 7, Gr. 8, and Gr. 9, respectively). For 8 weeks, all animals except those in Gr. 3 were administered 1 mL each of treatment via oral gavage once a day in the morning. Gr. 3 was subcutaneously injected with 100 U/mL NPH Humulin (Eli Lilly Asia Inc., Thailand branch, Bangkok) [59] in the same fashion for 8 weeks. The blood glucose was measured using a glucose test kit. Their rats’ body weights and the appearance of their eyes were recorded and observed daily [60,61].

#### 2.3.1. Biochemical Assays

At the end of the experiment, the rats were sacrificed by being injected with an overdose of 50 mg/kg BW (i.p.) of thiopental sodium. Their blood was collected for the determination of insulin, glucose, BUN, creatinine, uric acid, cholesterol, HDL, triglyceride, total protein, total bilirubin, direct bilirubin, AST/SGOT, ALT/SGPT, and alkaline phosphatase [61].

#### 2.3.2. Vascular Effect Test in Diabetic Rats

Once the rats were sacrificed, their thoracic aortas were extracted as soon as possible. The blood vessels were cut to a length of 3–4 mm and placed in Krebs bicarbonate solution containing 118 mM sodium chloride (NaCl), 25 mM sodium bicarbonate (NaH_2_CO_3_), 11 mM glucose, 1.6 mM CaCl_2_, 4.7 mM potassium chloride (KCl), 1.2 mM potassium dihydrogen phosphate (KH_2_PO_4_), and 1.18 mM magnesium sulfate (MgSO_4_) at 37 °C [62,63]. Each blood vessel was suspended on a hook connected to an isometric force transducer attached to a PowerLab data acquisition system (ADInstruments, Sydney, Australia) with a resting force of 1 g. The blood vessel was left at this suspended state until reaching equilibrium for at least 1 h, and during the experiment, the Krebs bicarbonate solution was freshly changed every 15 min.

- Effects on the contraction of the thoracic aorta

The maximum contraction force of the blood vessel from each group of rats was measured after 80 mM KCl solution was added [62]. The arterial constriction ability of the blood vessels from normal and diabetic rats from all treatments was studied by adding 10^−10^–10^−4^ M phenylephrine (PE) into the thoracic aorta organ bath to achieve the cumulative dose response. The experimental results were expressed as the percentage contraction compared to the maximum contraction.

- Effects on thoracic aorta relaxation

For the relaxation ability of the blood vessels from normal and diabetic rats from all groups, the arteries of all rats were induced to moderately contract with 10^−6^ M PE followed by the addition of 10^−10^–10^−4^ M acetylcholine (Ach) to observe the cumulative dose response [62]. The results were reported in the same manner as in Effects on the contraction of the thoracic aorta.

#### 2.3.3. Histological Study of the Pancreas

The heart, lung, liver, kidney, adrenal gland, pancreas, spleen, prostate gland, seminal vesicle, epididymis, and testis were dissected and weighed. The pancreas histology and immunohistochemistry procedures were based on Kim et al. [64], with some modifications. A portion of the pancreas was immersed in 10% formalin and sectioned to no more than 3 mm thick with a surgical blade. After that, the sectioned samples were prepared for biopsy by washing, dehydrating, clearing or dealcoholizing, and then being embedded in a paraffin block. Then, they were sliced with a microtome to a thickness of 5 microns. The sliced tissue was floated in a water bath at 43–45 °C, attached to a glass slide, and left to air dry at room temperature. The sample slides were dried again at 56 °C to melt the residual paraffin followed by staining with hematoxylin and eosin (H&E) to reveal details of the tissue and cells. The slides were mounted with a mounting medium and covered with cover slips. They were examined under a microscope with a magnification of 400 times.

#### 2.3.4. Immunohistochemistry Study of Beta and Alpha Cells of the Pancreas

The pancreas was dissected, weighed, and immersed in 4% paraformaldehyde at 4 °C overnight. The tissues were then embedded in paraffin and then sectioned into 5 µm thick for immunohistochemistry staining. The sectioned samples were deparaffined and rehydrated with washing buffer. To inhibit the activity of endogenous peroxidase, a 0.3% hydrogen peroxide solution was added, followed by normal goat serum, and incubated with monoclonal mouse antibody (MMA)–insulin or MMA–glucagon at 1:1000 at 4 °C overnight. After washing with phosphate-buffered saline (PBS) buffer, the tissue samples were treated with biotinylated rabbit anti-mouse immunoglobulin G, washed with PBS buffer, and incubated with streptavidin peroxidase reagent before adding a few drops of 3,3-diaminobenzidine tetrahydrochloride. The sections were then counterstained with hematoxylin, dehydrated, mounted with mounting medium, covered with a coverslip, and examined under a 200× magnification microscope [64]. An area of ten islets per pancreas in each group was measured. The Image J program was used to determine the number of beta cells per islet (insulin-positive area), glucagon per islet (glucagon-positive area), insulin intensity, and glucagon intensity in the pancreas (Wayne Rasband, Research Services Branch, National Institute of Mental Health, Bethesda, MD, USA). The effect of insulin intensity was reported as the difference in mean pixel intensity between normal control rats. Glucagon intensity was calculated by comparing the mean pixel intensity of the diabetic rats to the mean pixel intensity of the control diabetic rats, which was adjusted to 100% [65].

### 2.4. Statistical Analysis

The data are expressed as mean ± standard error of the mean (SEM). Statistical analysis was carried out using SPSS 16.0 for Windows (SPSS Inc., Chicago, IL, USA) and one-way analysis of variance (ANOVA). The least significant difference (LSD) test was used to compare differences in treatment means within a sampling period. *p*-values lower than 0.05 were considered statistically significant.

## 3. Results and Discussion

### 3.1. Diabetes Induction by STZ

Table 1 shows the blood sugar levels and body weight of STZ-treated rats compared to those if the normal or control group. The blood sugar was increased from 83 to 526 mg/dL during the 4 weeks of STZ treatment, whereas the body weight of the treatment group was reduced to 189 g in the 4th week. The methyl nitrosourea group in STZ is the active group that binds to the cell membrane and passes into the beta cells via the mechanism of action of STZ. It destroys beta-cell DNA through the alkylation process by releasing nitrosourea in the O6 position of guanine [66,67]. STZ has also been shown to induce nitric oxide (NO), an islet cell destroyer, as well as DNA destruction [68]. Superoxide anion radicals produced by STZ inhibiting the Krebs cycle [69] contribute to the destruction of DNA strands and reduce mitochondrial oxygen consumption. Xanthine oxidase (XOD) is then activated, resulting in the production of superoxide radicals (O_2_^−^), hydrogen peroxide (H_2_O_2_), and hydroxyl radicals (OH-). NO and the superoxide radical (O_2_^−^) can react separately and/or together to form peroxynitrite (ONOO^−^), which can also damage DNA strands [70]. When a DNA strand is damaged, poly (ADP-ribose) synthetase is activated, and NAD is the precursor to DNA repair, decreasing NAD in beta cells [71]. This decrease in NAD results in beta-cell necrosis, abnormal beta-cell function, and a decrease in proinsulin synthesis, causing beta cells to release less insulin and ultimately leading to diabetes [72].

In this study, STZ was injected into rats to induce diabetes. The rats lost weight in the second, third, and fourth weeks. Since the body cannot use the glucose produced by carbohydrate metabolism for energy, muscle protein and fat are burned instead, resulting in muscle atrophy. Subsequently, the body reduces the amount of accumulated fat, causing diabetic rats to lose weight. The diabetic rats’ blood glucose levels increased in the first, second, third, and fourth weeks, accompanied by diabetic patient-like symptoms, including excessive water intake (polydipsia), excessive urination (polyuria), and binge eating (polyphagia). This is due to STZ’s unique ability to destroy pancreatic beta cells. The beta cells produce the hormone insulin, which plays an important role in facilitating the entry of glucose into cells. Cells use glucose to metabolize and generate energy. Since STZ reduced or eliminated insulin production in pancreatic beta cells, cells cannot metabolize glucose for energy, resulting in a higher accumulation of glucose in the blood, as in diabetes [73].

**Figure 1:**
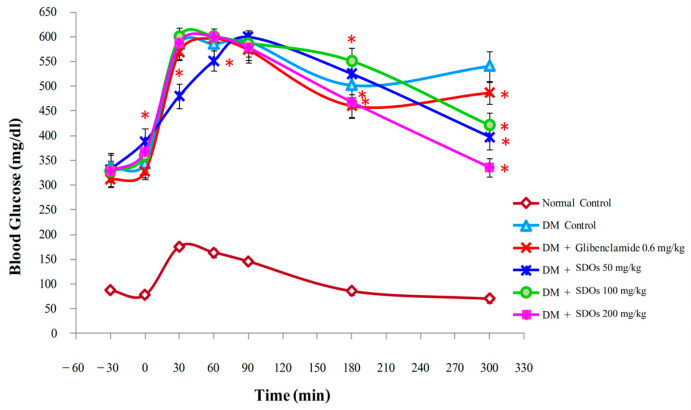
Effects of glibenclamide and SDOs at different concentrations on blood sugar levels in diabetic rats. Values are expressed as mean ± SD; six rats per group; * *p* < 0.05: significant compared with diabetic control.

An OGTT was used to determine the acute effects and optimal quantification of SDOs on blood glucose reduction in diabetic rats with acute hyperglycemia (Figure 1). After administering a glucose solution at a concentration of 5 g/kg BW to normal control rats, the blood glucose level increased over time before gradually decreasing until it reached the initial value at 180 and 300 min, respectively. Blood glucose levels in diabetic rats increased after 30 min but then decreased slightly after 60, 90, and 180 min. At 300 min, the blood glucose levels of all SDO- and glibenclamide-treated rats were significantly lower than those of control diabetes rats, but they remained above the pre-glucose infusion baseline, similar to the previous study by Chika and Bello [56]. SDOs at 200 mg/kg had the best glucose-lowering effect. Diabetic rats treated with SDOs demonstrated a dose-dependent decrease in blood sugar. The study showed that SDOs at doses of 50, 100, and 200 mg/kg effectively lowered blood sugar in diabetic rats.

### 3.2. Effects of SDO Administration on Diabetic Rats

All diabetic rats weighed significantly less than normal control rats (Table 2). Diabetic rats given insulin, SPI, and SDOs at concentrations of 100 and 200 mg/kg gained significantly more weight than control diabetic rats. All diabetic rats had significantly higher blood sugar levels than normal rats. However, diabetic rats treated with insulin, glibenclamide, SPI, and SDOs at all concentrations had significantly lower blood sugar levels than control diabetic rats. Diabetic rats in all groups had significantly lower serum insulin levels than normal rats. The diabetic rats given insulin and 200 mg/kg SDOs had significantly higher serum insulin levels than the control diabetic rats. Insulin and glibenclamide are two commonly used drugs to treat diabetic patients. This study found that insulin, but not glibenclamide, helped diabetic rats gain weight significantly more than control diabetic rats. This is consistent with the findings of Moglia et al. [59] and Naowaboot et al. [74]. The drugs helped diabetic rats gain weight compared to control diabetic rats, but the difference was not statistically significant. The findings of the study revealed that BSA, a standardized protein, had no effect on increasing the body weight of diabetic rats, similar to Zhu et al. [50], who studied the effect of oligopeptides extracted from the skin of marine salmon on type 2 diabetic rats compared to diabetic rats given 3 g/kg BW of BSA.

In this study, we used SPI to demonstrate the activity of only the protein components of soy. SPI has been shown in previous studies to help diabetic rats gain weight, like the findings of Zuo et al. [30] and Sites et al. [75], who discovered that SPI could increase body weight. There was a statistically significant increase in body weight in diabetic rats given SDOs compared to control diabetic rats since SDOs increase insulin levels in diabetic rats’ sera (Table 2). As a result, the body can absorb various nutrients and use glucose to generate energy, eliminating the need to metabolize protein from muscles and fat, resulting in increased body weight.

STZ-induced diabetes in rats revealed a statistically significant increase in blood sugar levels compared to those in normal control rats (Table 2). The blood sugar level was in the 300–350 mg/dL range. STZ destroys pancreatic beta cells, resulting in decreased insulin production and secretion and increased blood sugar levels. However, diabetic rats given insulin, glibenclamide, SPI, and SDOs at concentrations of 50, 100, and 200 mg/kg had significantly lower blood sugar levels than the control diabetic rats. This is consistent with the findings of Moglia et al. [59] and Naowaboot et al. [74], who discovered that when diabetic rats were administered insulin, blood sugar levels decreased similarly to those of normal control rats [76]. Glibenclamide has a hypoglycemic effect on rats with mild or moderate diabetes. At this point, pancreatic beta cells retain the ability to produce insulin. In this study (Table 2), we discovered lower serum insulin levels. Glibenclamide can also lower blood sugar levels, and its effects may extend beyond the pancreas. It improves glucose delivery to cells as well as glucose utilization by target cells, resulting in lower blood sugar levels [77]. SPI has been shown to lower blood sugar levels in STZ-induced diabetic rats, possibly by increasing insulin secretion [78] or reducing insulin resistance. SDOs at a concentration of 200 mg/kg had the greatest effect because they significantly increased serum insulin levels (Table 2). This study’s findings suggest that SDOs can help reduce diabetes.

The findings (Table 2) revealed that all groups of diabetic rats had significantly lower serum insulin levels than the normal control rats. STZ destroys beta cells, resulting in reduced insulin production and secretion. However, treating diabetic rats with insulin and 200 mg/kg of SDOs resulted in significantly higher serum insulin levels than those of the control diabetic rats. This could be due to SDOs’ ability to stimulate beta cells to increase insulin secretion, help beta cells to recover and return to normal function, or increase the number of beta cells. When diabetic rats were treated with glibenclamide and SPI, their serum insulin levels did not differ from those of control diabetic rats. Despite stimulating insulin secretion from beta cells, glibenclamide in this study did not increase insulin levels. This could be attributed to the severity of the condition. At this point, the pancreas’ beta cells must have been mostly destroyed, resulting in insufficient insulin production and secretion. SPI, according to this research, did not increase insulin levels either. This contradicts findings that SPI at 200 g/kg BW could stimulate insulin secretion from beta cells. The difference in SPI concentrations used in this study, 200 mg/kg (1000 times lower) versus 200 g/kg BW in Lee’s study [78], may explain the differences in results.

The weights of internal organs in diabetic rats differed significantly from those in control rats (Table 3). They weighed less, except for the liver and kidneys of the diabetic rats, which were heavier. Hypoinsulinemia may cause an increased input of fatty acids into the liver, leading to triglyceride accumulation which could explain the study’s observed significant increase in liver weight [79]. Similar findings are reported in the study of Lee et al. [80].

### 3.3. Effects on Serum Lipid Levels

STZ-induced diabetes in rats increased total cholesterol and triglyceride levels while decreasing HDL levels (Table 4), consistent with the findings of Muruganandan et al. [81]. Under normal conditions, insulin causes the lipoprotein lipase enzyme to hydrolyze triglycerides [82]. In diabetes, researchers discovered that a lack of insulin inhibits the stimulation of the lipoprotein lipase. As a result, triglycerides in the blood are elevated, particularly in diabetic rats. When diabetic rats were treated with insulin and SDOs at doses of 100 and 200 mg/kg, triglycerides were significantly lower than in control diabetic rats. SPI significantly increased HDL levels when compared to control diabetic rats but had no statistically significant effect on total cholesterol or triglyceride levels. This is consistent with Lee’s [78] findings, which showed that SPIs reduced total cholesterol and triglyceride levels while increasing HDL levels in STZ-induced rats. When diabetic rats were administered SDOs, triglyceride levels decreased, while HDL levels increased significantly compared to those in control diabetic rats. Overall, HDL levels in diabetic rats treated with glibenclamide, BSA, SPI, and SDOs differed significantly from those of control diabetic rats. Total cholesterol levels were highest in the control diabetic rats but decreased in the treatment groups, with only insulin differing significantly from the diabetic group. Cholesterol levels in the SDO-fed groups decreased as SDO doses increased. This could be because SDOs raise serum insulin levels (Table 2), causing HDL to rise and triglycerides to fall significantly, particularly at 100 and 200 mg/kg. This suggests that SDOs could reduce the risk of arteriosclerosis [83].

### 3.4. Effects on Clinical Chemistry Values

All diabetic rat groups had similar high BUN levels (Table 4), except the insulin-treated group, which showed a significant difference from the control diabetic rats. Diabetic rats treated with BSA and SDOs at a concentration of 200 mg/kg had lower creatinine levels than the normal control rats. Normal Wistar rats exhibit BUN 11–25 mg/dL and creatinine 0.2–0.7 mg/dL [84,85]. All groups fed protein had elevated BUN levels but lower creatinine levels than the normal control group. This can be caused by diabetes and the high-protein diet treatment. Diabetic rats may lose their muscle mass at a faster rate than the normal control group, as indicated by the rapid decrease in body weight. However, when the diabetic rats were given enough insulin, BUN and creatinine levels along with body weight were like those of the normal control group. Uric acid levels in diabetic rats were high in all groups as an indication of their impaired glucose metabolism from diabetes or purine containing diet. However, lower uric acid levels and increased body weight in diabetes rat groups could be an indication that their impaired glucose metabolism improved, as in those diabetic rats given insulin, SPI, and SDOs at 100 and 200 mg/kg. The levels of blood urea nitrogen (BUN) and uric acid indicate kidney function. STZ, which has a negative effect on the kidneys, was discovered to raise BUN and uric acid levels in diabetic rats. As a result, these values increased, which is consistent with the findings of Eidi et al. [61] However, diabetic rats in the group that received insulin and SDOs at 200 mg/kg showed a statistically significant decrease in BUN and uric acid levels when compared to those of control diabetic rats, indicating that insulin and SDOs at 200 mg/kg can help reverse diabetes-induced kidney function impairment.

The insulin-treated group had significantly lower cholesterol than the control diabetic group (Table 4). However, diabetic rats administered glibenclamide, BSA, SPI, and SDO had significantly higher cholesterol than normal rats but non-significantly lower cholesterol than control diabetic rats. The control diabetic rats and the insulin-treated group had the lowest levels of HDL. The remaining treated diabetic groups had significantly higher HDL levels than the control diabetic rats, bringing them closer to the normal group. Triglyceride levels in diabetic rats were significantly higher in all groups than in the normal control rats. However, the groups that received insulin and SDOs at 100 and 200 mg/kg had significantly lower triglyceride levels than the control diabetic rats. The rest of the diabetic groups differed significantly from the normal control, similar to the study of Tunali et al. [86]. All diabetic rats had lower total protein levels than normal control rats (Table 4). Nevertheless, the diabetic rats administered insulin, glibenclamide, and SDOs at 100 and 200 mg/kg had significantly higher total protein levels than the control diabetic rats getting them closer to the normal control group. All diabetic rats had significantly lower albumin levels than the normal control rats; however, when treated with insulin, glibenclamide, BSA, SPI, and SDOs at all concentrations, the albumin levels were significantly increased to levels higher than those in control diabetic rats, similar to a previous report by Park et al. [87]. Higher serum albumin levels may indicate that diabetic groups have improved in terms of not receiving enough nutrients, having liver and kidney problems, or suffering from an inflammatory disease caused by diabetes. Diabetic rats treated with SDOs at a dose of 50 mg/kg had significantly lower total bilirubin levels than normal control rats, whereas diabetic rats treated with BSA and SPI had higher total bilirubin levels. Diabetic rats treated with SPI had higher levels of direct bilirubin compared to control, normal, and diabetic rats. The diabetic rats treated with BSA and SPI may have had a higher rate of red blood cell breakdown than the others, resulting in significantly higher total and direct bilirubin levels as a byproduct of liver metabolism passing through the intestine [88]. AST and ALT enzyme levels are indicators of liver function. In diabetic rats, high levels of AST and ALT may be due to liver damage, which allows a large amount of these enzymes to leak out of the liver cells and into the bloodstream [89,90]. This demonstrates that STZ damages the liver, consistent with Eidi et al. [61]. Diabetic rats in all groups had higher levels of alkaline phosphatase than control rats, indicating a liver problem or bone disorder. When diabetic rats were given insulin and SDOs at doses of 100 and 200 mg/kg, alkaline phosphatase levels dropped by 26–40%, which means that the liver or bones worked better [91].

**Figure 2:**
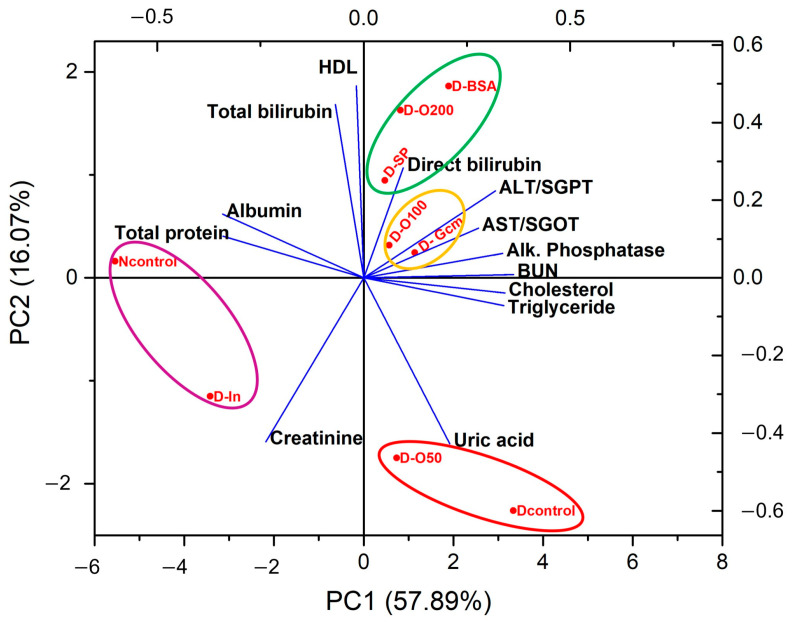
PCA of blood chemistry values from all rat groups. The groups in the same color circle are closely related in blood chemistry results.

To obtain a good picture of the correlation between rat groups, blood chemistry values were analyzed with PCA, and the results show that rats can be divided into four groups, as shown in Figure 2. The normal control group is closely related to the diabetic rats given insulin (purple circle). Diabetic rats in the control group and those treated with 50 mg/kg SDOs are close to each other based on uric acid (red circle). The diabetic rats treated with BSA, SDOs at 200 mg/kg, and SPI can be placed in the same group (green circle), while those given glibenclamide and SDOs at 100 mg/kg (yellow circle) are very closely related to each other.

**Figure 3:**
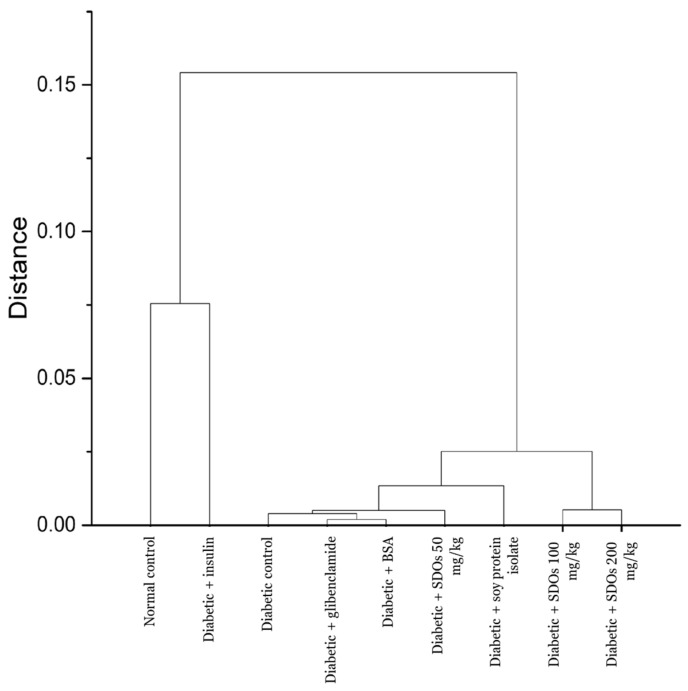
Hierarchical cluster analysis (HCA) of blood chemistry values from all rat groups.

A hierarchical clustering analysis (HCA) based on blood test results was performed to illustrate the differences and similarities among the rat groups, Figure 3. We can categorize them into four clusters using a distance of 0.025. Cluster 1 comprises normal rats that distinctively differ from others. Cluster 2 is made up of insulin-treated diabetic rats. The blood chemistry data from this group differ from those of diabetic rats in other groups. Cluster 3 consists of diabetic control rats and diabetic rats treated with glibenclamide, BSA, SPI, and SDOs at a dosage of 50 mg/kg. SDO-treated diabetic groups at 100 and 200 mg/kg make up the last cluster. This clearly illustrates the differences and similarities between and within all groups.

### 3.5. Effects on Eye Appearance

**Figure 4:**
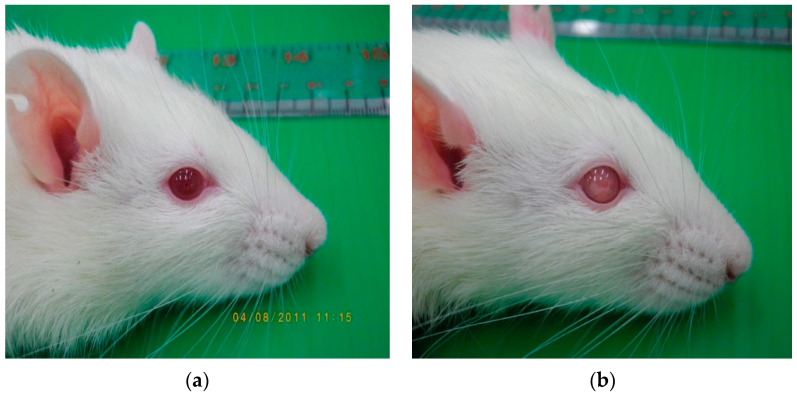
Rat eyes: (**a**) normal rat; (**b**) diabetic rat.

Normal rats had bright and clear eyes, while the diabetic control group showed cloudiness in both eyes (Figure 4 and Table 5). In contrast, insulin-treated diabetic rats lacked clear eyes but did not show cloudiness. Two diabetic rats, treated with glibenclamide and SDOs at 100 mg/kg, showed normal eyes. In the groups that received BSA, SPI, and 50 mg/kg SDOs, only one diabetic rat exhibited normal vision in both eyes. Three diabetic rats treated with SDOs at a dose of 200 mg/kg showed normal vision in both eyes. This is because prolonged high blood sugar levels affect capillaries throughout the body and alter the blood vessel walls of the retinae in the eyes. The endothelium thickens (hyperplasia), resulting in small blood vessel blockages (microvascular occlusion) [89,92], as well as the loss of pericytes, which surround blood vessels in the retina. Pericytes play a crucial role in stabilizing the structure of blood vessels [90]. The loss of pericytes weakens the blood vessels and causes fluid leakage (microvascular leakage) from them [93], leading to retinal edema. When this occurs for an extended period, it leads to the formation of new blood vessels, bleeding, clouding of the vitreous fluid inside the eye, fibrosis, and pulling of the retina, which may eventually result in a cataract. Diabetes retinopathy is a side effect of diabetes [94]. In this study, we considered the rats’ eyes cataractous when the lens appears cloudy white to the naked eye, following the guidelines from Chen et al. [95]. Insulin-treated diabetic rats had some eyes that appeared unclear but were not considered cataractous. This could be because insulin helps to lower blood sugar levels to near-normal levels, reducing the risk of complications. According to this study, increasing SDO doses, particularly those at 200 mg/kg, resulted in a 50% reduction in cataracts [91]. The findings suggest that SDOs help lower blood sugar levels by increasing blood insulin levels, thereby delaying this complication.

### 3.6. Effects on Blood Vessels in Diabetic Rats

Diabetes is associated with blood vessel hardening, kidney impairment, and cardiovascular disease caused by improper blood vessel relaxation and contraction. In this study, we investigated how each treatment affected the contraction–relaxation function of the thoracic aorta over a long period of diabetes, as coronary artery disease (CAD) or coronary heart disease (CHD) is commonly associated with the diabetic population.

#### 3.6.1. Effects on Contraction of the Thoracic Aorta

**Figure 5:**
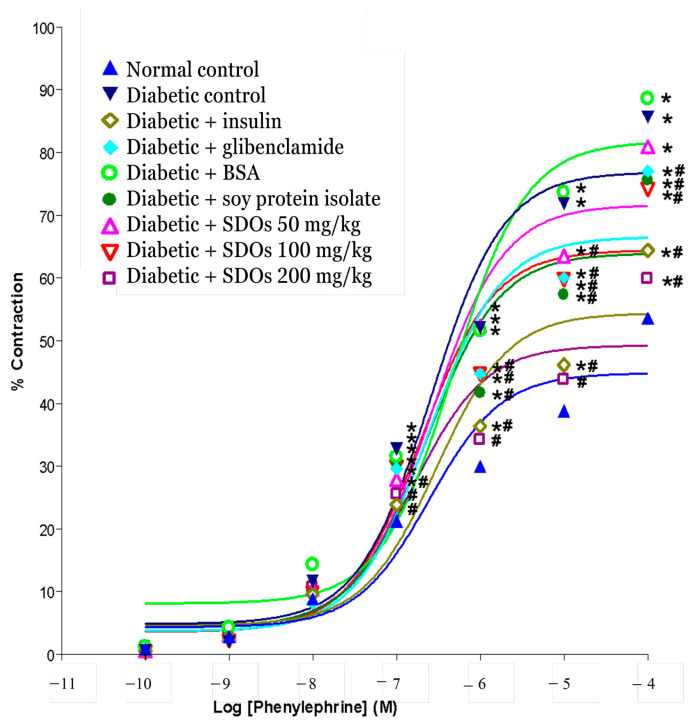
Effects of various treatments on the contraction of arteries: thoracic aorta. Values are expressed as mean ± S.E.M.; six rats per group. * *p* < 0.05 compared to normal control group. # *p* < 0.05 compared to diabetic control group.

To compare the effect of SDOs on thoracic aortic contraction–relaxation to that of other treatments, phenylephrine (PE) was applied to the blood vessels. The cumulative dose of PE at concentrations ranging from 10^−10^ to 10^−4^ M caused increased artery contraction in all groups of rats (Figure 5). PE at a dose of 10^−7^ M or higher caused the arteries of control diabetic rats and the BSA-treated group to contract significantly more than the arteries of normal rats and diabetic rats treated with insulin, glibenclamide, SPI, and SDOs at all three concentrations. SDOs at 200 mg/kg were able to help the arteries contract significantly less than glibenclamide and SPI but not significantly differently from the normal control rats.

For this study, we injected STZ into rats to induce diabetes and then administered various substances for 8 weeks. We separated the thoracic aorta at the end of the experiment to study the effect on artery contraction and relaxation. Stimulation of the arteries of control diabetic rats caused greater contraction than in normal rats (Figure 5), which was consistent with previous research by Abebe et al. [92]. The exact way the response works is not known, but it could be caused by an endothelium that cannot handle phosphoinositide (PI) metabolism [89], calcium channels that are more sensitive [92], or adrenergic agonists that are more likely to work [90]. Furthermore, oxidative stress increases because of increased free radical generation and decreased antioxidant capacity [93]. Glycoxidation and lipid peroxidation cause this oxidative stress, which can eventually lead to a variety of diabetes complications. Oxidative stress could increase diacylglycerol–protein kinase activity in the aortae of diabetic rats [96], leading to impaired endothelial function, increased PI turnover, and increased arterial contraction. This is caused by changes in calcium channel activity in the arteries of rats induced by STZ. As a result, oxidative stress in diabetic rats may cause increased arterial contraction as well as impaired endothelial activity [97].

#### 3.6.2. Effects on Relaxation of the Thoracic Aorta

**Figure 6:**
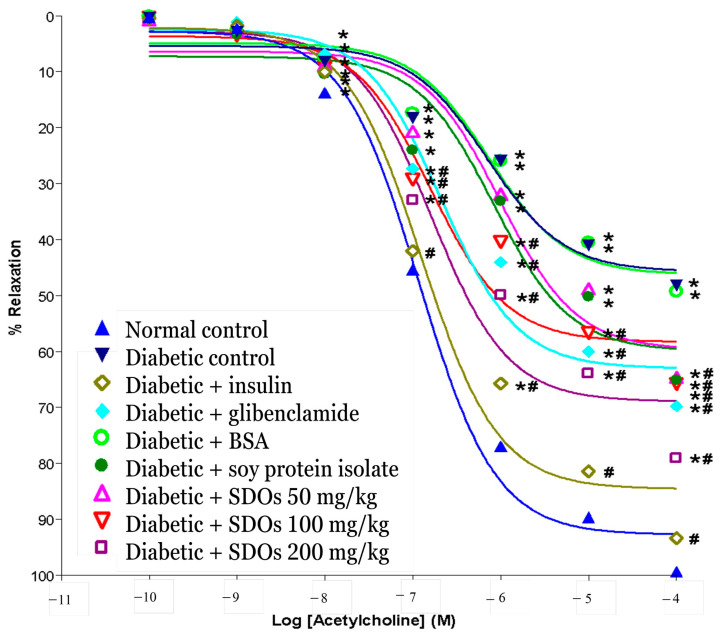
Effects of various treatments on the relaxation of arteries: thoracic aorta. Values are expressed as mean ± S.E.M.; six rats per group. * *p* < 0.05 compared to normal control group. # *p* < 0.05 compared to diabetic control group.

It was discovered that administering Ach at cumulative doses of 10^−10^–10^−4^ M caused artery relaxation in all groups of rats. After stimulating contraction with PE (10^−6^ M), arterial relaxation increased as Ach increased, and Ach concentrations of 10^−8^ M and higher increased arterial relaxation. When compared to that in normal control rats, diabetic artery relaxation was significantly reduced in both control and BSA-treated diabetic rats. Diabetic rats treated with insulin, glibenclamide, SPI, and SDOs at all three concentrations had significantly greater artery relaxation than control diabetic rats. In terms of artery relaxation, SDOs at 200 mg/kg outperformed glibenclamide and SPI. Furthermore, as illustrated in Figure 6, control diabetic rats exhibited less relaxation in response to Ach. According to Kamata et al. [98] and Oyama et al. [99], endothelium-dependent relaxation in response to agonists such as Ach is impaired in the aorta of diabetic rats. Oxidative stress destroys the endothelium, resulting in decreased vascular relaxation and endothelial NO production and secretion. As a result, diabetic rats’ vascular smooth muscle responses to NO are reduced [100]. In this study, we investigated the role of SDOs in preventing diabetes-related arterial dysfunction. SDOs were discovered to be effective in diabetic rats, preventing both the reduction in endothelium-dependent relaxation and the increase in vasoconstrictor responses. The concentration of 200 mg/kg produced the best results and outperformed SPI at the same concentration. It is possible that SDOs reduce oxidative stress in diabetic rats, but this was found to be significantly different from the control diabetic rats and the normal group, like diabetic rats treated with insulin and glibenclamide. This might be attributed to SDOs’ antioxidant properties [101], and this mechanism of oxidative stress reduction has been described in previous research by Samanta et al. [102].

### 3.7. Study of Histopathology of the Pancreas

**Figure 7:**
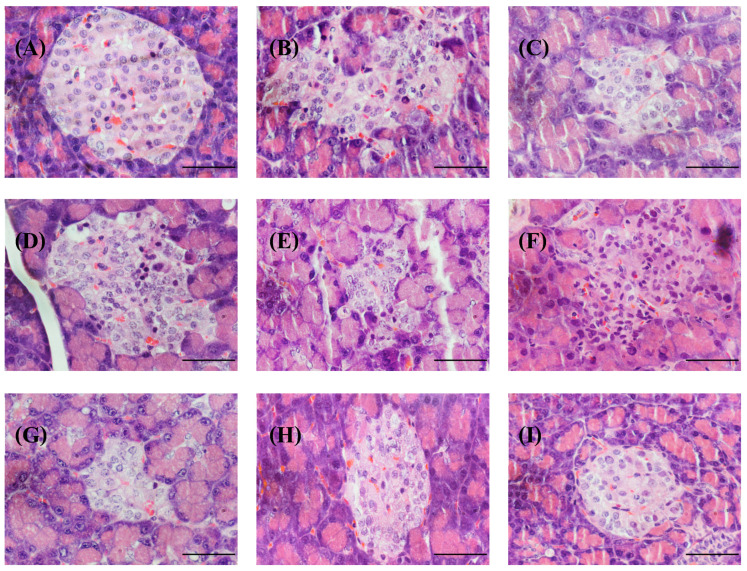
Histopathology of islets of Langerhans in the pancreas of normal control and diabetic rats after exposure to various substances for 8 weeks (magnification 400 times; scale bar 50 microns). (**A**) Normal control; (**B**) diabetic control; (**C**) diabetic + insulin; (**D**) diabetic + glibenclamide; (**E**) diabetic + BSA; (**F**) diabetic + SPI; (**G**) diabetic + SDOs 50 mg/kg; (**H**) diabetic + SDOs 100 mg/kg; (**I**) diabetic + SDOs 200 mg/kg.

All normal control rat pancreas islets of Langerhans stained with H&E had normal and similar histopathological features, including a round or oval shape. The pancreas and exocrine glands were clearly separated and consisted of polygonal cells of similar sizes that were densely packed together. The nucleus was round, blue in color, and clearly visible. The cytoplasm evenly distributed the pink eosin dye throughout the cell, with capillaries inserted between the cells. Figure 7A shows sporadic red blood cells within the Langerhans islets. Control diabetic rats had islets of Langerhans histopathological features that differed from those of normal control rats, as follows: The size of the Langerhans islets shrank, and some small and large cells had irregular shapes. The nucleus was oval, dark blue in color, with some of it was obscured and unable to be seen clearly. The cytoplasm was unevenly pink, with vacuoles scattered throughout some cells (Figure 7B), indicating that the cells were wilting and increasing the space between them. Some areas had white spots, indicating cell death, because some cells were larger than their neighbors. Smooth cell edges indicated cellular swelling [103]. Diabetic rats given insulin, glibenclamide, BSA, or SPI showed the same histopathology as diabetic control rats (Figure 7C, Figure 7D, Figure 7E, and Figure 7F, respectively). This means that the substances have no effect on cell regeneration in the Langerhans islets. However, Figure 7G–I clearly shows that diabetic rats given SDOs experienced fewer and milder symptoms of various conditions, particularly at 200 mg/kg, where islets appeared round like those of normal control rats, indicating that SDOs could restore some damaged beta cells in the islets of Langerhans without causing additional damage to pancreatic cells, similar to previous research by Zhao et al. [104] using green silk cocoon shells.

### 3.8. Immunohistochemistry Study of Beta and Alpha Cells of the Pancreas

#### 3.8.1. Effects on Beta-Cell Distribution

**Figure 8:**
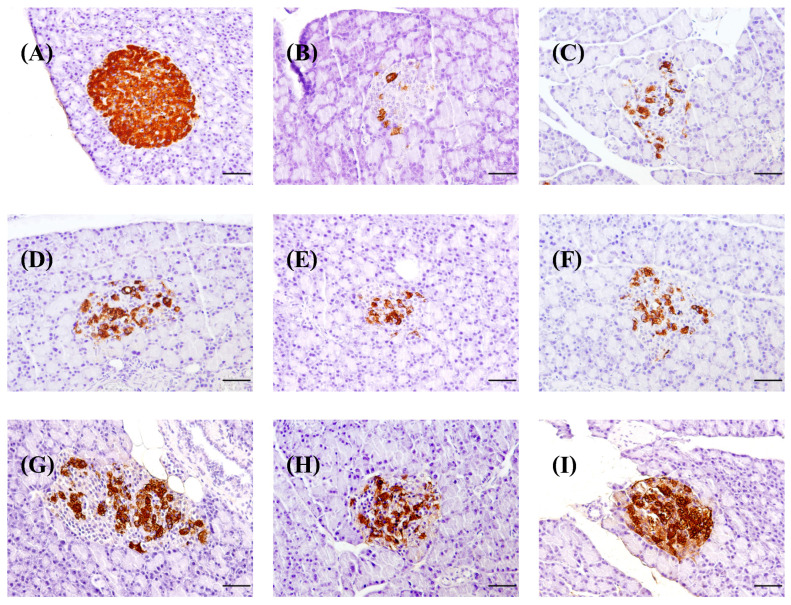
Distribution of beta cells in the pancreas of normal control and diabetic rats after exposure to various substances for 8 weeks (magnification 200 times; scale bar 50 µm). (**A**) Normal control; (**B**) diabetic control; (**C**) diabetic + insulin; (**D**) diabetic + glibenclamide; (**E**) diabetic + BSA; (**F**) diabetic + SPI; (**G**) diabetic + SDOs 50 mg/kg; (**H**) diabetic + SDOs 100 mg/kg; (**I**) diabetic + SDOs 200 mg/kg.

**Figure 9:**
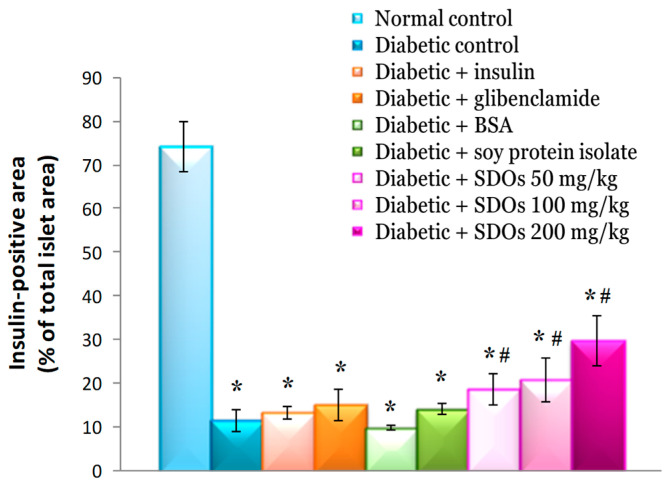
Area of beta cells in the islet of Langerhans (insulin-positive area) of normal control and diabetic rats after exposure to various substances for 8 weeks. Values are expressed as mean ± SD; six rats per group. * *p* < 0.05 compared to normal control group. # *p* < 0.05 compared to diabetic control group.

**Figure 10:**
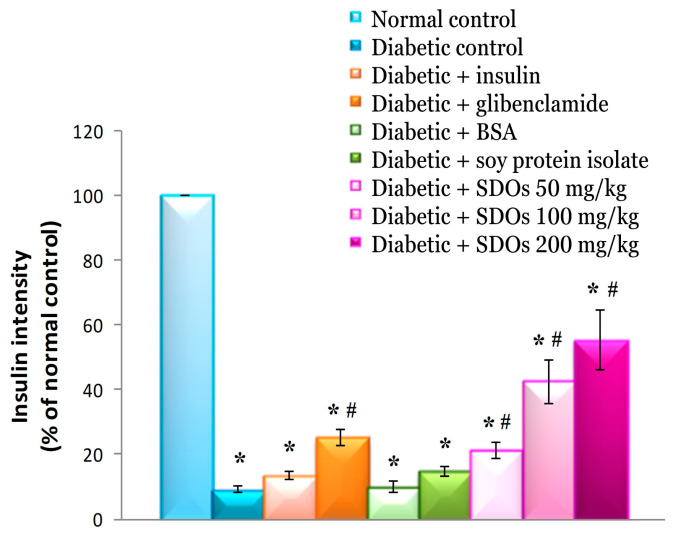
Insulin intensity in the pancreas of normal control and diabetic rats after exposure to various substances for 8 weeks. Values are expressed as mean ± SD; six rats per group. * *p* < 0.05 compared to normal control group. # *p* < 0.05 compared to diabetic control group.

This technique involved staining the pancreas with anti-insulin and anti-glucagon antibodies. It demonstrated that in normal rats, the islets of Langerhans had more beta cells than alpha cells. The nuclei of these cells were stained blue, with uniformly brown cytoplasm surrounding them. Beta cells were densely packed in the center of the islets, while alpha cells were found near the edges of the Langerhans islets (Figure 8A), which is consistent with Ahmed et al.’s [105] findings. Ahmed et al. [105] discovered that inducing diabetes in rats alters the distribution and number of beta and alpha cells. In diabetic rats, beta cells had blue nuclei with only a few brown granules in the cytoplasm. Most cells in the cytoplasm are unmarked. When compared to the control group of normal rats, the control diabetic rats had significantly less beta-cell area (Figure 8A,B), insulin-positive area (Figure 9), and insulin intensity (Figure 10), indicating that many beta cells had been destroyed and died, resulting in a decrease in insulin levels in the pancreas. STZ destroys pancreatic beta cells by damaging their DNA strands, causing hyperglycemia in rats and leading to diabetes [106]. Diabetic rats administered insulin, glibenclamide, BSA, SPI, and SDOs at all doses showed some improvement via intense brown staining of the beta cells (Figure 8C–I), particularly those given SDOs at all doses, which showed a larger area of brown staining than the other diabetic groups.

#### 3.8.2. Effects on Size of the Area of Beta Cells in the Islet of Langerhans (Insulin-Positive Area)

In diabetic rats, the area of beta cells per islet of Langerhans was 11.46 ± 2.47%, which was significantly lower than that in normal rats (74.15 ± 5.78%) and roughly the same as that in diabetic rats treated with BSA (9.79 ± 0.68%) (Figure 9). The area of beta cells per islet of Langerhans in diabetic rats fed SPI was 14.09 ± 1.25%, which was higher than that in control diabetic rats but not statistically significant. When compared to control diabetic rats, diabetic rats treated with SDOs at doses of 50, 100, and 200 mg/kg had significantly larger beta-cell area sizes per islet of Langerhans, measuring 18.51 ± 3.51%, 20.73 ± 5.10%, and 29.73 ± 5.70%, respectively. It was also discovered that increasing the SDO concentration increased the number of beta cells per islet of Langerhans. This suggests that SDOs could aid in the proliferation of beta cells in diabetic rats. The reduction in beta-cell area size and insulin intensity in diabetic rats treated with insulin and BSA, which was also observed in control diabetic rats, is consistent with the findings of Latha and Daisy [103], who administered 3 U/kg insulin to diabetic rats, and of Zhu et al. [50], who used BSA on diabetic rats. STZ destroyed beta cells, resulting in a substantial reduction in their number. Glibenclamide did not increase beta-cell area in diabetic rats, but it did significantly increase insulin intensity (Figure 10) when compared to that in the control diabetic group. Although glibenclamide has no effect on beta-cell count, it can help the pancreas to produce more insulin. This is because glibenclamide, a sulfonylurea that stimulates beta cells to secrete more insulin [77], can lower blood sugar levels. There was no increase in beta-cell space size or insulin intensity in diabetic rats fed SPI. Our study found no effect of SPI on pancreatic beta-cell count or insulin levels (Table 2). This contradicts Lee’s [78] findings that SPI at 200 g/kg could stimulate increased insulin secretion by beta cells. The contradictory results may be explained by the 1000-fold lower concentration of SPI used in this study, which was only 200 mg/kg.

#### 3.8.3. Effects on Insulin Intensity in the Pancreas

Diabetic rats in the control group had significantly lower insulin staining intensity than normal rats (9.12 ± 0.97% per 100.00 ± 0.00%), and the values were similar to those for diabetic rats receiving BSA and insulin (9.95 ± 1.96% and 13.56 ± 1.39%, respectively) (Figure 10). Diabetic rats fed SPI had an insulin intensity of 14.72 ± 1.27%, which was comparable to that in control diabetic rats. Diabetic rats treated with glibenclamide and SDOs at 50, 100, and 200 mg/kg had significantly higher insulin intensity levels compared to those in control diabetic rats (25.19 ± 2.50%, 21.30 ± 2.54%, 42.60 ± 6.70%, and 55.44 ± 9.06%, respectively). Diabetic rats treated with SDOs demonstrated an increase in insulin intensity as the SDO concentration increased. The experimental results show that glibenclamide and SDOs at all doses can increase insulin levels in the pancreas. When diabetic rats were treated with all three SDO concentrations, both beta-cell area size and insulin intensity increased, with SDOs at 200 mg/kg having the greatest effect. SDOs have been shown to increase beta-cell count and insulin output. It is possible that SDOs could aid in beta-cell regeneration and/or cell proliferation. Furthermore, SDOs may stimulate beta cells, causing increased insulin secretion. As a result, the pancreas produces more insulin (Table 2). Wang et al. [107] found that pancreatic endocrine cells can proliferate following STZ-induced diabetes. The proliferation of beta cells after destruction by STZ is associated with the replication of the pre-existing surviving beta cells in order to compensate for the reduced number and respond to the functional demand [108]. However, our experiment found that diabetic rats treated with SDOs still had fewer beta cells than normal control rats.

#### 3.8.4. Effects on Distribution of Alpha Cells

**Figure 11:**
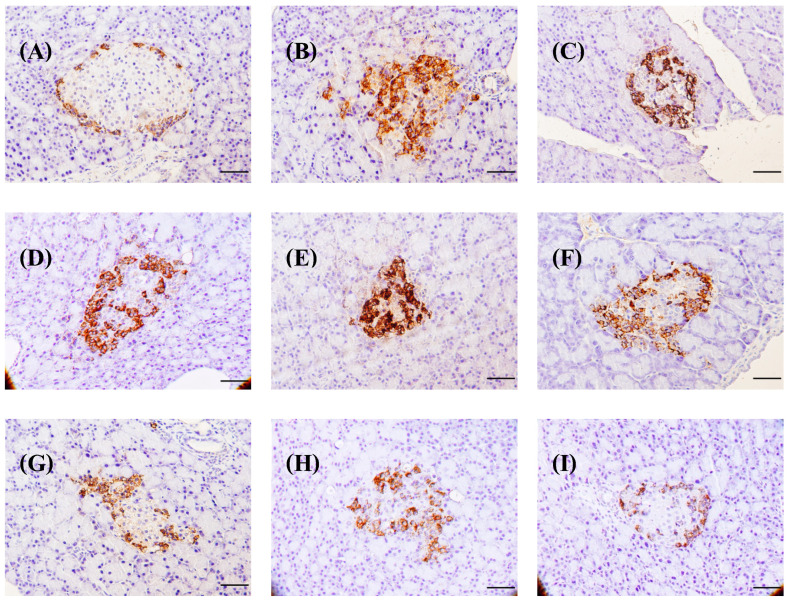
Characteristics of the distribution of alpha cells in the pancreas of normal control and diabetic rats after exposure to various substances for 8 weeks (magnification 200×; scale bar 50 µm). (**A**) Normal control; (**B**) diabetic control; (**C**) diabetic + insulin; (**D**) diabetic + glibenclamide; (**E**) diabetic + BSA; (**F**) diabetic + SPI; (**G**) diabetic + SDOs 50 mg/kg; (**H**) diabetic + SDOs 100 mg/kg; (**I**) diabetic + SDOs 200 mg/kg.

In normal control rats, immunohistochemistry staining with an anti-glucagon antibody revealed that alpha cells were arranged around the edge of the islets of Langerhans, with blue-stained nuclei of hematoxylin and uniform brown granules in the cytoplasm (Figure 11A). In diabetic rats, alpha cells with blue nuclei and brown granules in the cytoplasm were found in the islets of Langerhans (Figure 11B). Diabetic rats treated with insulin, glibenclamide, BSA, and SPI had the same distribution of alpha cells as control diabetic rats (Figure 11C–F). However, alpha cells in diabetic rats given SDOs had blue nuclei with brown granules scattered around the islets of Langerhans, resembling those in normal control rats (Figure 11G–I).

#### 3.8.5. Effects on Size of the Area of Alpha Cells in the Islet of Langerhans (Glucagon-Positive Area)

**Figure 12:**
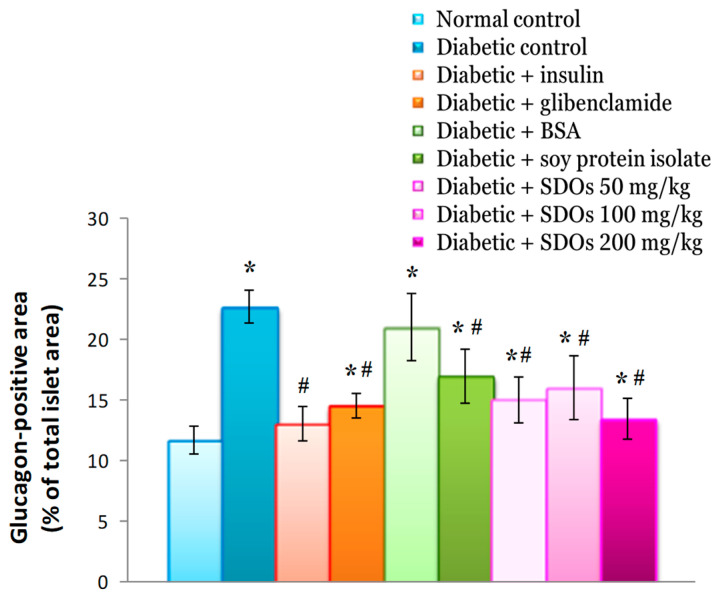
Area of alpha cells in the islet of Langerhans (glucagon-positive area) of normal control and diabetic rats after exposure to various substances for 8 weeks. Values are expressed as mean ± SD; six rats per group. * *p* < 0.05 compared to normal control group. # *p* < 0.05 compared to diabetic control group.

Control diabetic rats had an area of alpha cells per islet of Langerhans of 22.70 ± 1.36%, significantly higher than that of normal control rats (11.71 ± 1.14%) but similar to that of diabetic rats administered BSA (21.03 ± 2.80%) (Figure 12). In diabetic rats treated with insulin, glibenclamide, SPI, and SDOs at 50, 100, and 200 mg/kg, the area size of alpha cells per islet of Langerhans was 13.00 ± 1.39%, 14.52 ± 1.02%, 16.92 ± 2.22%, 15.01 ± 1.93%, 15.95 ± 2.62%, and 13.44 ± 1.68%, respectively. These values were statistically significant compared to those in diabetic control rats. It was also discovered that as SDO concentrations increased in diabetic rats, the number of alpha cells per islet of Langerhans decreased. Insulin, glibenclamide, SPI, and SDOs at all three concentrations were similarly found to reduce the number of alpha cells in diabetic rats.

#### 3.8.6. Effects on Intensity of Glucagon (Glucagon Intensity) in the Pancreas

**Figure 13:**
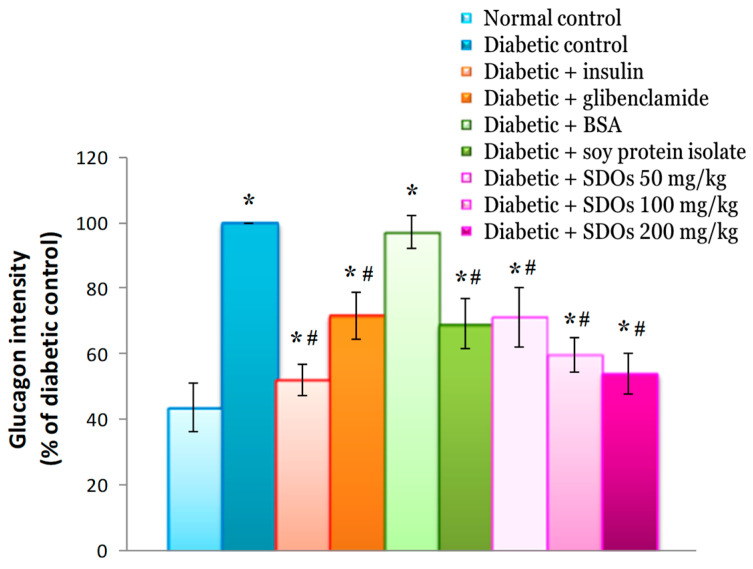
Glucagon intensity in the pancreas of normal control and diabetic rats after exposure to various substances for 8 weeks. Values are expressed as mean ± SD; six rats per group. * *p* < 0.05 compared to normal control group. # *p* < 0.05 compared to diabetic control group.

Diabetic control rats had significantly higher glucagon staining intensity than normal control rats (100.00 ± 0.00% to 43.67 ± 7.50%), with values comparable to those of diabetic rats administered BSA (97.20 ± 4.97%) (Figure 13). The glucagon intensity in diabetic rats treated with insulin, glibenclamide, SPI, and SDOs at concentrations of 50, 100, and 200 mg/kg was 52.19 ± 4.74%, 71.70 ± 7.20%, 69.17 ± 7.71%, 71.17 ± 8.99%, 59.66 ± 5.26%, and 53.99 ± 6.11%, respectively, which was significantly lower than that in control diabetic rats. The intensity of glucagon in diabetic rats administered SDOs decreased as the concentration increased. This study discovered that insulin, glibenclamide, SPI, and SDOs at all three concentrations could help lower pancreatic glucagon levels. The distribution of alpha cells in the islets of Langerhans was found to be central in diabetic rats (Figure 11B–I). The destruction of beta cells in the middle of the islets of Langerhans resulted in their shrinkage. As a result, the alpha cells repositioned themselves in the center of the Langerhans islets. Furthermore, it was discovered that an increase in both alpha cell number and glucagon content resulted in an increase in alpha cell area and glucagon intensity in the pancreas of diabetic rats. Low levels of insulin caused by damaged beta cells may be a factor for the increase in alpha cell number and glucagon intensity. When STZ destroys beta cells, the body responds by increasing alpha cells, which can transform into new beta cells, along with glucagon, a crucial hormone for the formation and differentiation of beta cells. Therefore, the increase in alpha cells and glucagon intensity may help assist in the recovery of new beta cells in diabetic rats [109]. On the other hand, diabetic rats with higher insulin levels showed fewer alpha cells and lower levels of glucagon. This could be because insulin binds to insulin receptors on alpha cells, stopping the intracellular cAMP-PKA pathway and lowering glucagon secretion in alpha cells [110]. This can explain why rats with low insulin levels would exhibit increases in both glucagon and alpha cells. When administered at 200 mg/kg, SDOs help beta cells grow back, but this can raise serum insulin levels in around 38% of normal control rats and 50% of insulin-treated diabetic rats. Insulin can still lower the activity of glucagon and the number of alpha cells, just like it can in the other two groups. Despite the loss of most beta cells, insulin-treated diabetic rats still showed decreased levels of glucagon and alpha cells. This suggests that serum insulin levels have a greater influence on alpha cell proliferation and glucagon activity than the damaged mass of beta cells. An abnormal beta-cell insulin secretion pattern causes prolonged hyperglycemia and/or impaired inhibition of alpha cell glucagon secretion [104]. When diabetic rats received insulin, glibenclamide, SPI, and SDOs at three concentrations, the area of alpha cells and the amount of glucagon in the pancreas significantly decreased compared to those in control diabetic rats. This demonstrates that the substances have the ability to lower blood sugar, resulting in a reduction in alpha cells and glucagon intensity in the pancreas. However, we found only SDOs to cause significant changes in the insulin-positive area and insulin intensity, indicating their ability to aid in beta-cell recovery from STZ damage.

Previous research demonstrated that peptides control glucose metabolism through different pathways, including enhancing insulin secretion, improving the body’s sensitivity to insulin, and inhibiting the activities of key enzymes [111]. In our digestive system, three key enzymes are involved in glucose metabolism, including α-amylase, α-glucosidases, and dipeptidyl peptidase 4 (DPP-IV) [112]. Pancreatic α-amylase, which is produced by the pancreas and secreted into the duodenum, is a crucial enzyme that breaks down dietary carbohydrates like starch into simple monosaccharides [113]. Consequently, these are further degraded by α-glucosidases, which are located in the brush border of the small intestine. α-glucosidases selectively hydrolyze monosaccharides at terminal (1→4)-linked α-glucose residues to single α-glucose molecules that can be absorbed and enter the bloodstream [114]. Therefore, the inhibition of α-amylase and α-glucosidase retards the final stages of carbohydrate digestion, consequently delaying the entry of glucose into the circulation and lowering post-prandial glucose levels [115]. Interestingly, α-amylase and α-glucosidase activity can be inhibited by sericin peptides; the small molecule exhibited the greatest inhibitory activity [116]. The mechanism of inhibition can be explained based on the study of Xie et al. [117]. The inhibition was a reversible and non-competitive type. When sericin peptides bound to α-D-glucosidase at one site, mainly through hydrogen bonding and van der Waals forces, they altered the conformation of α-D-glucosidase. As the concentration of the peptides increased, the overall secondary structure of α-D-glucosidase exhibited an increase in β-turns and random coils and a decrease in α-helices and β-sheets. Through the conformational change, the enzyme’s catalytic efficiency is reduced. Owing to the abovementioned mechanism, sericin peptides inhibit α-amylase and α-glucosidase activity by altering their conformation, which then reduces the enzymes’ efficiency in breaking down starch, resulting in lower glucose in the blood. This is unlike acarbose, an α-glucosidase inhibitor that exhibits competitive inhibition by binding to the oligosaccharide binding site of α-glucosidase or α-amylase, which then prevents the binding of the oligosaccharide substrate [118]. Nevertheless, sericin peptides and acarbose still exert similar effects on lowering glucose in blood.

The SDOs in this study are mixed oligopeptides with MW < 3 kDa composed of fractions that have an IC_50_ for DPP-IV inhibitory activity between 1.3 and 1.4 mM (unpublished data). Their stability and bioactivity for DPP-IV inhibitory effects remained unchanged after four hours of in vitro GI digestion with pepsin and pancreatin when compared to sericin. Following in vitro blood plasma hydrolysis of the most active peptide fractions, DDP-IV inhibitory activity decreased slightly [54]. According to our research on yellow silk cocoons, SDOs may function similarly to incretin hormones such as glucagon-like peptide-1 (GLP-1) and gastric inhibitory peptide (GIP), causing pancreatic b cells to release insulin. Upon absorption into the bloodstream [119], plasmin, a human plasma serine protease, degrades SDOs into smaller peptides while still inhibiting DPP-IV. Similar to GLP-1 (1–37) amide, it is degraded to GLP-1 (7–36) and (7–37) amides in the blood and neutralized by DPP-IV [120,121]. SDOs suppressed glucagon activity, like GLP-1, and reduced pancreatic β-cell apoptosis while increasing proliferation. This may be due to their antioxidant activity and competition for DPP-IV, which left more GLP-1 available in the blood circulatory system, resulting in enhanced incretin hormone activity (GLP1, GIP), leading to improved beta-cell health and insulin secretion. Previous research has reported that GLP-1 stimulates proliferation, survival, and differentiation in both in vitro and in vivo models. As a result, GLP-1 resolves both the defect in insulin secretion and the decline in b-cell mass, both of which contribute to the deterioration of b-cell function in T2DM [122]. Furthermore, yellow silkworms only feed on organic mulberry leaves containing 1-deoxynojirimycin (1-DNJ), an iminosugar that has been developed as a new class of diabetic medications [13] To increase absorbability for GLP-1 receptor agonists, salcaprozate sodium or sodium N-[8-(2-hydroxybenzoyl) amino] caprylate is required [121]. Yellow silkworms naturally bind sericin to lutein in the form of protein-binding lutein, like the structure of lutein in the human retina, making SDOs partially hydrophilic in nature [123]. This may help to increase their absorption. Efforts are being made to increase sericin’s efficiency through conjugation with compounds such as polyphenol. Omar et al.’s studies [124,125] found that the covalent binding of flavonoids and phenolic compounds altered the conformation (secondary and tertiary structure) and thermal behavior of sericin, increased its surface hydrophobicity and morphology, and had a positive effect on sericin’s emulsifying activity. Furthermore, sericin hydrolysate demonstrated higher antioxidant and anti-inflammatory activity than native sericin hydrolysate [125]. As a result of their improved emulsifying, antioxidant, and anti-inflammatory properties, sericin-phenolic conjugates may enhance SDO’s antidiabetic effects. Based on our results and available research on sericin peptides, we hypothesize that SDOs could have hypoglycemic effects in the small intestine by inhibiting the activities of a-amylase and a-glucosidase. Once absorbed into the blood circulatory system, they would compete with GLP-1 and GIP for DPP-IV, which in turn would help lower the blood sugar level via the mechanism of active GLP-1 and/or GIP.

## 4. Conclusions

Results have shown that SDOs help diabetic rats gain weight, lower blood sugar levels, and increase serum insulin concentration, possibly due to the increased number of beta cells and insulin production in the pancreas, while decreasing alpha cells and glucagon levels. SDOs help serum lipid levels return to normal by lowering triglyceride levels while increasing HDL levels, with a slight decrease in total cholesterol levels, indicating that they can help reduce the risk of arteriosclerosis (atherosclerosis). Treatment with SDOs, especially at 200 mg/kg, may improve the liver and kidney functions of diabetic rats, as evidenced by the decrease in AST and ALT enzymes, BUN, and uric acid compared to levels in diabetic control rats. Although SDOs can reduce the blood sugar level, they are not yet completely effective in preventing cataracts in diabetic rats. Research has shown that only SDOs at 100 and 200 mg/kg, along with glibenclamide, can delay this complication by reducing cataracts by 50%. In diabetic rats, SDOs inhibit both the decrease in endothelial-dependent relaxation and the increase in vasoconstrictor responses, particularly at a concentration of 200 mg/kg, possibly by reducing oxidative stress. More research needs to be conducted to investigate this mechanism of action. Based on our findings, SDOs can reduce the deterioration effect of diabetes through a variety of mechanisms, including stimulating insulin secretion from beta cells, aiding in beta-cell recovery, and increasing the number of beta cells. As a result, serum insulin levels rise, which in turn lowers blood sugar levels.

## Figures and Tables

**Table 1:** Blood glucose and body weight of normal control and diabetic rats after 4 weeks.

Data	Week	Groups	
		Control (6 Rats)	Diabetic Rats (6 Rats)
Blood glucose (mg/dL)	Initial	84.50 ± 5.50	83.33 ± 5.35
	1	96.17 ± 7.68	356.83 ± 33.11 *
	2	105.83 ± 6.74	371.67 ± 24.06 *
	3	111.17 ± 5.34	495.67 ± 51.28 *
	4	115.33 ± 8.12	526.50 ± 27.48 *
Body weight (g)	Initial	196.83 ± 5.67	199.00 ± 6.48
	1	231.00 ± 6.36	230.83 ± 9.28
	2	278.33 ± 9.40	219.50 ± 6.38 *
	3	312.17 ± 15.73	204.33 ± 15.50 *
	4	333.00 ± 12.93	189.33 ± 7.79 *

Values are expressed as mean ± SD, * *p* < 0.05 compared to normal control group.

**Table 2:** Body weight, blood glucose, and insulin levels of normal control, diabetic, and diabetic rats with treatments for 8 weeks.

Groups									
Data	Normal Control	Diabetic Control	Diabetic + Insulin	Diabetic + Glibenclamide	Diabetic + BSA	Diabetic + SPI	Diabetic + SDOs (mg/kg)		
							50	100	200
Initial body weight (g)	259.17 ± 2.04	258.33 ± 7.53	258.50 ± 8.19	260.83 ± 6.65	259.80 ± 6.38	261.00 ± 3.37	253.33 ± 5.16	259.17 ± 4.92	260.00 ± 3.16
Final body weight (g)	453.33 ± 8.16	246.83 ± 25.42 *	404.00 ± 35.51 *#	260.67 ± 26.82 *	245.33 ± 20.20 *	284.50 ± 28.17 *#	265.83 ± 26.42 *	298.00 ± 32.89 *#	290.50 ± 37.68 *#
Initial blood glucose (mg/dL)	70.33 ± 1.86	320.17 ± 17.46 *	332.17 ± 21.18 *	319.33 ± 19.95 *	330.50 ± 18.96 *	334.33 ± 24.02 *	336.33 ± 25.32 *	328.33 ± 25.01 *	330.17 ± 25.79 *
Final blood glucose (mg/dL)	72.67 ± 3.27	369.50 ± 15.27 *	115.00 ± 11.70 *#	336.00 ± 19.46 *#	362.67 ± 16.34 *	329.50 ± 15.97 *#	335.83 ± 15.63 *#	320.00 ± 12.70 *#	306.50 ± 9.16 *#
Insulin level (ng/mL)	3.50 ± 0.81	0.25 ± 0.08 *	2.62 ± 0.50 *#	0.24 ± 0.06 *	0.28 ± 0.04 *	0.29 ± 0.05 *	0.30 ± 0.12 *	0.36 ± 0.12 *	1.33 ± 0.09 *#

Values are expressed as mean ± SD; six rats per group. * *p* < 0.05 compared to normal control group. # *p* < 0.05 compared to diabetic control group.

**Table 3:** Internal organ weights of normal control, diabetic, and diabetic rats with treatments for 8 weeks.

Parameters	Normal Control	Diabetic Control	Diabetic + Insulin	Diabetic + Glibenclamide	Diabetic + BSA	Diabetic + SPI	Diabetic + SDOs (mg/kg)		
							50	100	200
Heart (g)	1.24 ± 0.18	0.89 ± 0.10 *	1.06 ± 0.07 *#	0.86 ± 0.06 *	0.74 ± 0.05 *#	0.84 ± 0.11 *	1.00 ± 0.14 *	1.01 ± 0.11 *	0.96 ± 0.11 *
Lung (g)	1.41 ± 0.17	1.14 ± 0.10 *	1.26 ± 0.10 *	1.15 ± 0.10 *	1.03 ± 0.05 *	1.11 ± 0.05 *	1.26 ± 0.14 *#	1.24 ± 0.07 *	1.13 ± 0.07 *
Liver (g)	9.79 ± 0.91	10.96 ± 1.25 *	11.83 ± 0.87 *	10.00 ± 0.56	9.82 ± 0.66 #	10.41 ± 0.77	10.38 ± 0.48	11.09 ± 0.69 *	10.72 ± 1.20
Kidney (g)	2.09 ± 0.16	2.82 ± 0.28 *	2.47 ± 0.19 *#	2.67 ± 0.17 *	2.70 ± 0.14 *	2.84 ± 0.10 *	2.71 ± 0.13 *	2.77 ± 0.09 *	2.63 ± 0.16 *
Adrenal gland (g)	0.11 ± 0.03	0.08 ± 0.01 *	0.08 ± 0.01 *	0.07 ± 0.01 *	0.07 ± 0.01 *	0.07 ± 0.01 *	0.08 ± 0.01 *	0.09 ± 0.01 *	0.07 ± 0.01 *
Pancreas (g)	0.64 ± 0.15	0.66 ± 0.17	0.63 ± 0.18	0.66 ± 0.16	0.50 ± 0.05 #	0.60 ± 0.08	0.49 ± 0.09 #	0.52 ± 0.06	0.57 ± 0.13
Spleen (g)	0.79 ± 0.11	0.54 ± 0.08 *	0.63 ± 0.05 *	0.45 ± 0.02 *	0.42 ± 0.09 *#	0.49 ± 0.05 *	0.52 ± 0.03 *	0.56 ± 0.08 *	0.53 ± 0.12 *
Prostate gland (g)	0.27 ± 0.05	0.23 ± 0.07	0.27 ± 0.02	0.18 ± 0.06 *	0.13 ± 0.05 *#	0.18 ± 0.05 *	0.13 ± 0.05 *#	0.12 ± 0.05 *#	0.17 ± 0.05 *
Seminal vesicle (g)	1.08 ± 0.28	0.54 ± 0.19 *	1.17 ± 0.11 #	0.37 ± 0.15 *	0.56 ± 0.19 *	0.58 ± 0.13 *	0.68 ± 0.17 *	0.77 ± 0.20 *#	0.49 ± 0.16 *
Epididymis (g)	1.55 ± 0.20	0.99 ± 0.20 *	1.35 ± 0.07 *#	0.95 ± 0.14 *	0.82 ± 0.13 *	1.01 ± 0.10 *	0.93 ± 0.12 *	1.17 ± 0.08 *	1.08 ± 0.25 *
Testis (g)	4.00 ± 0.30	3.43 ± 0.25 *	3.90 ± 0.15 #	3.32 ± 0.14 *	3.13 ± 0.19 *#	3.47 ± 0.10 *	3.46 ± 0.30 *	3.68 ± 0.09 *#	3.42 ± 0.23 *

Values are expressed as mean ± SD; six rats per group. * *p* < 0.05 compared to normal control group. # *p* < 0.05 compared to diabetic control group.

**Table 4:** Clinical chemistry values of normal control, diabetic, and diabetic rats with treatments for 8 weeks.

Parameters	Normal Control	Diabetic Control	Diabetic + Insulin	Diabetic + Glibenclamide	Diabetic + BSA	Diabetic + SPI	Diabetic + SDOs (mg/kg)		
							50	100	200
BUN (mg/dL)	17.42 ± 3.24	37.62 ± 11.29 *	22.62 ± 6.71 #	33.27 ± 7.30 *	34.72 ± 6.35 *	30.63 ± 9.13 *	31.42 ± 5.90 *	33.17 ± 7.51 *	31.77 ± 4.21 *
Creatinine (mg/dL)	0.67 ± 0.16	0.57 ± 0.21	0.60 ± 0.24	0.51 ± 0.20	0.45 ± 0.06 *	0.56 ± 0.10	0.58 ± 0.26	0.49 ± 0.16	0.42 ± 0.08 *
Uric acid (mg/dL)	1.64 ± 0.22	3.35 ± 0.87 *	2.69 ± 0.56 *	2.96 ± 0.66 *	2.07 ± 0.23 #	3.07 ± 0.44 *	3.41 ± 0.91 *	2.96 ± 0.64 *	2.21 ± 0.34 #
Cholesterol (mg/dL)	43.67 ± 6.02	82.67 ± 19.99 *	49.67 ± 5.32 #	72.67 ± 10.19 *	72.50 ± 8.07 *	75.33 ± 9.81 *	80.33 ± 6.65 *	80.00 ± 8.15 *	73.00 ± 8.05 *
HDL (mg/dL)	51.12 ± 9.51	34.02 ± 2.46 *	37.00 ± 5.87 *	46.98 ± 7.64 #	53.68 ± 3.26 #	51.65 ± 6.30 #	51.43 ± 7.13 #	52.00 ± 6.30 #	51.28 ± 8.21 #
Triglyceride (mg/dL)	77.17 ± 11.67	180.50 ± 41.41 *	121.50 ± 44.44 *#	150.83 ± 38.13 *	164.50 ± 46.42 *	151.00 ± 28.40 *	145.17 ± 22.52 *	124.00 ± 32.53 *#	140.17 ± 24.24 *#
Total protein (g/dL)	6.48 ± 0.18	4.87 ± 0.48 *	5.91 ± 0.40 *#	5.44 ± 0.28 *#	5.05 ± 0.12 *	5.23 ± 0.25 *	5.13 ± 0.46 *	5.53 ± 0.56 *#	5.64 ± 0.44 *#
Albumin (g/dL)	3.24 ± 0.12	2.21 ± 0.57 *	3.04 ± 0.20 #	2.66 ± 0.25 *#	2.65 ± 0.02 *#	2.75 ± 0.21 *#	2.76 ± 0.18 *#	2.75 ± 0.14 *#	2.77 ± 0.21 *#
Total bilirubin (mg/dL)	0.61 ± 0.10	0.46 ± 0.16	0.70 ± 0.14	0.68 ± 0.26	0.74 ± 0.50 #	0.84 ± 0.18 #	0.34 ± 0.06 *	0.50 ± 0.21	0.61 ± 0.09
Direct bilirubin (mg/dL)	0.06 ± 0.05	0.09 ± 0.08	0.09 ± 0.02	0.11 ± 0.08	0.09 ± 0.04	0.19 ± 0.05 *#	0.05 ± 0.02	0.12 ± 0.07	0.10 ± 0.06
AST/SGOT (U/L)	78.17 ± 7.19	132.17 ± 38.88 *	64.17 ± 9.68 #	123.33 ± 28.59 *	130.67 ± 35.98 *	73.00 ± 9.53 #	97.67 ± 23.98 #	109.33 ± 26.03 *	129.50 ± 17.73 *
ALT/SGPT (U/L)	29.17 ± 5.19	64.33 ± 9.11 *	26.17 ± 4.26 #	50.83 ± 19.91 *	68.67 ± 19.22 *	47.17 ± 10.23 *#	48.17 ± 17.08 *#	57.50 ± 14.27 *	67.33 ± 11.74 *
Alk. phosphatase (U/L)	61.50 ± 9.91	309.83 ± 42.09 *	91.00 ± 8.20 #	303.17 ± 55.92 *	306.33 ± 46.80 *	297.83 ± 42.93 *	264.50 ± 31.58 *	185.33 ± 24.63 *#	228.17 ± 57.16 *#

Values are expressed as mean ± SD; six rats per group. * *p* < 0.05 compared to normal control group. # *p* < 0.05 compared to diabetic control group.

**Table 5:** Eye characteristics of normal control, diabetic, and diabetic rats with treatments for 8 weeks.

Groups	Normal Eyes *	Left-Eyed Cataract *	Right-Eyed Cataract *	Both-Eyed Cataract *
Normal control	6	-	-	-
Diabetic control	-	-	-	6
Diabetic + insulin	6	-	-	-
Diabetic + glibenclamide	2	1	-	3
Diabetic + BSA	1	-	1	4
Diabetic + SPI	1	1	-	4
Diabetic + SDOs 50 mg/kg	1	1	-	4
Diabetic + SDOs 100 mg/kg	2	-	1	3
Diabetic + SDOs 200 mg/kg	3	-	-	3

* Based on Chen et al.’s [95] guidelines.

## Data Availability

The original contributions presented in the study are included in the article, further inquiries can be directed to the corresponding author.
